# Evaluation of Peripheral Blood Concentrations of Phoenixin, Spexin, Nesfatin-1 and Kisspeptin as Potential Biomarkers of Bipolar Disorder in the Pediatric Population

**DOI:** 10.3390/biomedicines12010084

**Published:** 2023-12-29

**Authors:** Lena Cichoń, Artur Pałasz, Krzysztof M. Wilczyński, Aleksandra Suszka-Świtek, Anna Żmijowska, Ireneusz Jelonek, Małgorzata Janas-Kozik

**Affiliations:** 1 Department of Developmental Age Psychiatry and Psychotherapy, John Paul II Pediatric Centre in Sosnowiec, Faculty of Medical Sciences, Medical University of Silesia in Katowice, ul. Zapolskiej 3, 41-218 Sosnowiec, Poland; 2 Department of Histology, Faculty of Medical Sciences, Medical University of Silesia in Katowice, ul. Medyków 18, 40-752 Katowice, Poland

**Keywords:** bipolar disorder, neuropeptides, spexin, kisspeptin, nesfatin-1, phoenixin

## Abstract

There are some initial suggestions in the literature that phoenixin, spexin, nesfatin-1 and kisspeptin may play a role in the pathogenesis of affective disorders. Therefore, they may also be cautiously considered as potential diagnostic or predictive biomarkers of BD. This study aimed to evaluate the levels of the aforementioned neuropeptides in the peripheral blood of children and adolescents with bipolar. This study included 122 individuals: 67 persons with diagnosed bipolar disorder types I and II constituted the study group, and 55 healthy persons were included in the control group. Statistically significant differences in the concentrations of neuropeptides between the control and study groups were noted in relation to nesfatin-1 and spexin (although spexin lost statistical significance after introducing the Bonferroni correction). In a logistic regression analysis, an increased risk of bipolar disorder was noted for a decrease in nesfatin-1 concentration. Lower levels of nesfatin-1 seemed to be a significant risk factor for the development of bipolar disorder types I and II. Furthermore, the occurrence of bipolar disorder was associated with significantly elevated levels of spexin. None of the analyzed neuropeptides was significantly correlated with the number of symptoms of bipolar disorder.

## 1. Introduction

Bipolar disorder (BD) is a chronic, episodic mood disorder. During its exacerbations, patients experience manic/hypomanic symptoms (such as elevated/irritable mood, racing thoughts, decreased need for sleep, heightened self-esteem, increased talkativeness, psychomotor agitation, easy distraction, and engagement in activities with a high potential for causing harmful consequences) or depressive episodes (i.e., depressed mood, loss of interest, decreased ability to feel pleasure, excessive guilt, weight loss, sleep disorders, lack of energy, psychomotor agitation/slowness, lack of self-esteem, concentration problems, and suicidal thoughts/attempts). In addition, sometimes, they also exhibit positive symptoms [1,2]. BD is often accompanied by other mental disorders; in the pediatric population, these are, inter alia, anxiety disorders, attention deficit hyperactivity disorder and behavioral or eating disorders. The diagnosis of BD in children and adolescents is often difficult to make due to the clinical picture often being different from that in adults and the coexistence of various mental disorders. Delayed diagnosis and thus postponed treatment are associated with a worse prognosis, while “overdiagnosis” may be the cause of unnecessary pharmacotherapy, which has numerous side effects for young people [1]. Thus, in the diagnosis of BD, it would be helpful to find more objective markers that allow for a correct diagnosis to be made quickly.

In this context, it is interesting to note the involvement of neuropeptide signaling in the pathogenesis of some mental disorders, including BD [3,4]. This type of neurotransmission is based on a wide range of several hundred peptides that play a variety of roles both within the central nervous system and peripherally. Their main functions are primarily focused on modulating synaptic activity, while, peripherally, their role is similar to that of hormones. For this reason, they arouse great interest in neurology and psychiatry, where there are more and more reports on their potential involvement in the pathogenesis of neurodegenerative diseases or mood disorders. For example, in the available literature, there are studies of brain tissues of patients with, inter alia, BD, in whom a decrease in the expression of neuropeptide Y (NPY) in the prefrontal cortex was observed compared to a control group [5,6]. However, the level of NPY in the cerebrospinal fluid of people with BD who had made suicide attempts in the past was significantly lower than in subjects without self-aggressive behaviors. NPY levels were particularly reduced in those who committed suicide during the period of psychiatric follow-up. In addition, patients in this group had lower levels of NPY than those who had a history of suicide attempts and had not attempted suicide again. The obtained results indicate that the determination of NPY levels in PMR may prove helpful, for example, in estimating the risk of future suicidal self-aggressive behaviors [7]. There are also suggestions that oxytocin could be a potential marker of the course of BD [8]. In the case of orexins (highly pleiotropic neuropeptides), the results of the determinations are contradictory, as both a decrease and an increase in the level of orexin A in the serum of patients with BD have been observed [9,10].

Both BD and schizophrenia are entities that are currently understood in psychiatry primarily through the prism of monoaminergic neurotransmission. Interestingly, most neuropeptides are released in the brain together with monoamines, modulating their function and physiological effects. For this reason, in recent years, the attention of researchers in the context of mood disorders has focused on several specific neuropeptides, including phoenixin (PNX), spexin (SPX), nesfatin-1 and kisspeptin, which have a multidirectional spectrum of physiological activity. There are reports indicating, for example, their involvement in the mechanisms of anxiety or the pathogenesis of eating disorders [11,12]. PNX is a poorly understood regulatory factor, occurring in the form of two molecular forms: a shorter form (PNX-14) and a longer form (PNX-20). It was identified using bioinformatic methods and was later revealed to be expressed in the hypothalamus, amygdala and rat brainstem [13,14]. PNX is a ligand of the metabotropic receptor GPR173 showing wide distribution in numerous brain structures [15]. The administration of PNX-14 into the ventricles of the brain and the hypothalamus of adult mice produces a strong anxiolytic effect, as demonstrated by behavioral tests [16]. However, pioneering clinical trials in 2017 demonstrated a relationship between PNX levels and anxiety in humans [17]. The involvement of this neuropeptide in the regulation of food intake, memory, Alzheimer’s disease, inflammation, neuronal and microglial activity, energy metabolism and body fluid balance, and cardiovascular and microglial metabolism is also increasingly taken into account. In addition, an interaction between PNX and nesfatin-1 has been observed [18]. It is believed that nesfatin-1, a molecule with a length of 82 amino acids, may enhance the effects of PNX-14 on the release of reproductive hormones, such as lutropin, follicle-stimulating hormone and testosterone, in male rats [19]. Nesfatin-1 is secreted after post-translational cleavage from the NEFA/nucleobidine 2 precursor due to the activity of specific convertases PC2 and PC3/1 [20,21]. It plays an important role in the hypothalamic pathways that regulate food intake and energy homeostasis. It is expressed in several neurons of the forebrain, the posterior part of the brain, the brainstem and the spinal cord. The administration of nesfatin-1 significantly inhibits consumption behavior and reduces weight gain in experimental animals. Nesfatin-1 has also been implicated in other important brain functions, such as reproduction, sleep, cognition, and anxiety or stress-related responses [22]. It is also being considered as a biomarker helpful in assessing the severity of depression [23]. However, SPX (discovered thanks to in silico molecular modeling techniques) is an anorexigenic neuropeptide [24] and an alternative ligand of Gal2 and Gal3 galanine receptors [25,26]. Neurons expressing SPX are present in many regions of the brain, inter alia, in the hypothalamus, hippocampus, amygdala, cerebellum and brainstem [27]. It has been observed that the intraventricular administration of a compound that is a structural analog of SPX induces an anxiolytic effect in rats [24]. Studies have also linked SPX to glycemic regulation (through the inhibitory relationship between insulin and SPX), as well as to body weight regulation, while other reports point to the anxiolytic, antidepressant and antipsychotic effects of SPX-related pathways in the amygdala [28]. However, kisspeptin, as a ligand of the metabotropic Kiss-1R receptor (GPRS54), plays a primary role in the regulation of reproductive functions in mammals, regulating the release of gonadotropin (GnRH) by hypothalamic neurons [29]. Kisspeptin is expressed in numerous neuronal populations of the hypothalamus, hippocampus and amygdala [30]. This neuropeptide also modulates insulin secretion and regulates the body’s energy balance by influencing the process of food intake [31]. Kisspeptin also participates in the mechanisms underlying sexual behavior and affective processes, and it has antidepressant and anxiolytic effects [32].

Despite the accumulating number of basic investigations, clinical studies regarding the possible involvement of PNX, nesfatin-1, SPX and kisspeptin in the origin and course of BD are still scarce and strongly insufficient. There are some initial suggestions that all aforementioned pleiotropic neuropeptides may play a role in the pathogenesis of several mental disorders [16,17,22,23,24,32]; therefore, they may also be cautiously considered as potential diagnostic or predictive biomarkers of BD. In our study, we assumed that the levels of PNX, nesfatin-1, SPX and kisspeptin in pediatric patients with BD may significantly differ from those in healthy controls. Based on this hypothesis, we aimed to assess the neuropeptide concentrations in BD patients by comparing them with those in healthy controls. Furthermore, we aimed to determine the manic, depressive and productive symptoms of the BD group and their correlations with PNX, nesfatin-1, SPX and kisspeptin levels. The key purpose of this study was therefore to estimate the differences in the peripheral blood concentrations of neuropeptides PNX, SPX, nesfatin-1 and kisspeptin between patients with BD and healthy controls, taking into account the disease episode and clinical picture.

## 2. Materials and Methods

### 2.1. Materials

This study was conducted at the Department of Psychiatry and Psychotherapy of Developmental Age of the Medical University of Silesia in Katowice, located at the John Paul II Center for Child and Family Health in Sosnowiec, together with the Department of Histology and Embryology of the Medical University of Silesia in Katowice. Study participants were divided into two groups: (1) those with a diagnosis of BD types I and II and (2) those with a clinically excluded diagnosis of BD based on the DSM-5 diagnostic criteria. The subjects with BD were recruited from the patients of the Clinical Department of Psychiatry and Psychotherapy of the Developmental Age (DPPDA) of the John Paul II Child and Family Health Center in Sosnowiec Sp. z o.o., and the control group was recruited from the patients of the Children’s Mental Health Clinic and other branches of the John Paul II Children’s and Family Health Center in Sosnowiec, as well as from students at schools in the Silesian Voivodeship. All study participants were under 18 years of age. Informed consent to participate in the study was obtained (after providing information on the study) from both the parents and the participants themselves, and the data collected during the study were pseudonymized. Exclusion criteria included mood disorders caused by medical conditions and the use of psychoactive substances. In contrast, inclusion criteria in the control group included subjects under 18 years of age with a clinically excluded diagnosis of BD and other psychiatric disorders based on the DSM-5 diagnostic criteria and not receiving psychiatric treatment.

### 2.2. Biochemical Assessment and Biological Material Collection

In the study group, the concentrations of nesfatin-1, PNX, SPX and kisspeptin in peripheral vein serum were determined at the beginning of hospitalization. In addition, in patients who were still being treated at DPPDA after 6 weeks and gave their consent, the concentrations of PNX, SPX and kisspeptin were determined again. In the control group, the concentrations of nesfatin-1, PNX, SPX and kisspeptin were determined once. Venous blood was collected on a clot activator. In all patients, blood sampling was performed while fasting, and at 7:30 a.m., the blood collection procedure was carried out identically for all samples, under conditions as minimally stressful as possible. Subsequently, all samples were centrifuged, and the serum obtained was stored at –20 °C until the determinations were made. The measurements of nesfatin-1, PNX, SPX and kisspeptin were performed using enzyme-linked immunosorbent assays (ELISAs) according to the protocols provided by the manufacturers of the kits: a sandwich enzyme-linked immunosorbent assay for the quantitative measurement of kisspeptin in vitro in human serum, plasma and other biological fluids (sensitivity: <13.1 pg/mL; detection range: 31.2–2000 pg/mL); a high-sensitivity ELISA assay for the quantitative determination of human nesfatin-1 serum concentration (sensitivity: 7.81 pg/mL; detection range: 31.25–2000 pg/mL); an enzyme-linked immunosorbent assay (EIA kit) for the detection of human PNX (sensitivity: 0.07 ng/mL; range: 0.07–2.1 ng/mL); and an ELISA kit for the in vitro quantitative determination of serum SPX concentration in humans m.in. (sensitivity: 46.88 pg/mL; range: 78.13–5000 pg/mL).

In addition, in 12 patients with BD, an additional blood sample was taken during hospitalization and pharmacological treatment to determine the PNX, SPX and kisspeptin levels 6 weeks after the first blood sample was taken. Out of these 12 subjects, 10 were treated with quetiapine at a maximum dose of 800 mg per day. In addition, 7 of the 10 subjects treated with quetiapine during these 6 weeks had aripiprazole added to their treatment at an increasing dose of 30 mg per day. Furthermore, 1 patient was treated with aripiprazole monotherapy, and 1 patient was treated with olanzapine and valproic acid. All patients were also periodically treated with diazepam at a maximum dose of 15 mg during hospitalization. In all of them, a significant reduction in the severity of psychopathological symptoms was observed during the 6 weeks of treatment in the mental state study and during observation within the ward.

### 2.3. Symptoms Analysis

In addition, the profile of selected symptoms presented by patients during an exacerbation of BD was analyzed. The assessment of symptoms was conducted via psychiatric, and the assessed symptoms were divided into three categories (manic, depressive and productive symptoms), as shown in Table 1.

### 2.4. Statistical Analysis

A statistical analysis was performed using StatSoft Statistica v13.0 software. The assumed significance level of α = 0.05 was modified according to the Bonferroni correction when analyzing correlations of more than 3 variables. Verification of the distribution of the variables was performed using the Shapiro–Wilk test; however, all distributions differed from the normal distribution. In order to compare independent samples, the Mann–Whitney U test was used. The Wilcoxon character pair test was used in the comparison of dependent samples, and the Spearman rho correlation test was used in the analysis of relationships between quantitative variables. In addition, logistic regression was used in the analysis to assess the discriminatory capacity of the neuropeptides in the context of the diagnosis of BD.

### 2.5. Bioethical Committee Consent

This study was conducted with the consent of the bioethics committee of the Medical University of Silesia in Katowice on the basis of resolution PCN/0022/KB1/126/19.

## 3. Results

### 3.1. Group Characteristics

This study included 122 individuals with a mean age of 14.87 years (95% CI: 14.57–15.17; min: 11; max: 19; SD: 1.63). The study group included 67 subjects with a mean age of 14.82 years (95% CI: 14.48–15.16; min: 11; max: 17; SD: 1.36), and the control group included 55 subjects with a mean age of 14.92 years (95% CI: 14.4–15.4; min: 12; max: 19; SD: 1.91). The age difference between the groups was not statistically significant in the Mann–Whitney U test with *p* > 0.05. In Spearman’s correlation analysis, the age of the subjects correlated with the PNX and SPX concentrations with *p* < 0.05. In the study group, males constituted 22.4% of the group (*n* = 15), and in the control group, they constituted 55.3% of the group (*n* = 31); the difference was statistically significant in the χ2 test with *p* > 0.05. However, in terms of the neuropeptides tested, no significant effect of gender on their concentrations was observed in the study group or in the control group in the Mann–Whitney U test (*p* > 0.05). The mean age of onset of BD was 13 years (95% CI: 12.5–13.5; min: 11; max: 16).

### 3.2. Comparative Analyses

In the analyzed group of patients diagnosed with BD, 26 patients (38.8%) were hospitalized due to a depressive episode, and 41 patients (61%) were hospitalized due to a manic episode. The number of symptoms reported by patients with BD and their parents and observed within the ward was analyzed. On average, the study group had 5.77 (95% CI: 5.23–6.31) manic symptoms, 9.38 (95% CI: 8.63–10.11) depressive symptoms and 2.09 (95% CI: 1.68–2.5) productive symptoms. In total, an average of 16.9 symptoms were observed in each patient (95% CI: 15.75–18.09).

**Figure 1:**
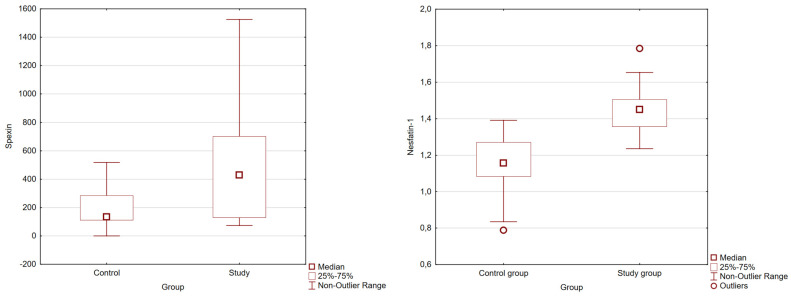
Differences in SPX and nesfatin-1 concentrations between the test and control groups (Mann–Whitney U test).

The average concentrations of the analyzed neuropeptides in the study and control groups are presented in Table 2. The statistically significant results are presented in Figure 1.

A comparison of concentrations between the patients hospitalized for a depressive episode and the patients hospitalized for a manic episode was undertaken. The results of the analysis are presented in Table 3.

**Figure 2:**
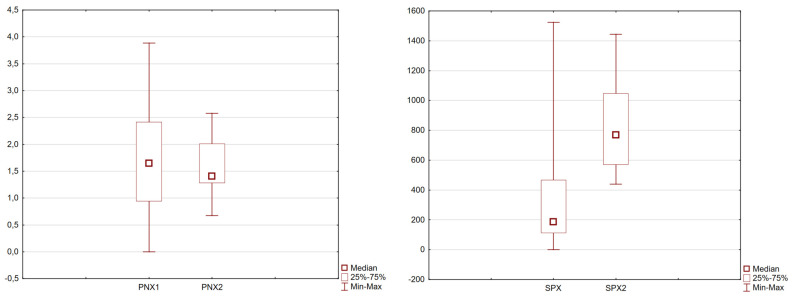
Differences in SPX and PNX concentrations before and after the 6-week pharmacotherapy period (*p*-value < 0.05 Wilcoxon pair test).

Finally, the effects of the pharmacotherapy on the concentrations of the neuropeptides after 6 weeks of treatment were analyzed. The obtained results are presented in Table 4 and Figure 2.

### 3.3. Correlation Analyses

Subsequently, Spearman’s correlation analysis of the relationship between the concentrations of the studied neuropeptides in the entire study population (study and control groups) was performed. The results are presented in Table 5.

Subsequently, Spearman’s correlation analysis was performed between the number of symptoms from each category and the concentrations of the analyzed neuropeptides, without obtaining statistically significant correlations at the assumed level of significance; therefore, the analysis using the Bonferroni correction was abandoned.

An analysis of Spearman’s rho correlation between the neuropeptide concentrations and the number of observed symptoms was also performed. It was noted that the concentration of SPX was significantly dependent on the number of manic symptoms with rho = −0.33 (*p* < 0.00625 **). In addition, no statistically significant relationships were observed between the number of symptoms and the concentrations of the neuropeptides studied.

### 3.4. Logistic Regression

A logistic regression analysis was also performed, in which only nesfatin-1 obtained statistical significance, with the odds ratio of qualifying to the control group equaling OR = 3.1 × 10^−16^ (95% CI: 0–0.007). The remaining neuropeptides did not achieve statistical significance in the analyzed model.

## 4. Discussion

The discussion of the results obtained should begin by considering the relevance of new concepts concerning mental disorders in general, including bipolar disorder, to the results of scientific research. Currently, in psychiatry, there is a shift in the perception of diagnostic units as dichotomous phenomena towards the concept of a “spectrum” or even a “continuum” of symptoms within the entire population [33]. This is clearly visible, for example, in the case of autism spectrum disorders or schizophrenia spectrum disorders. However, in the case of affective disorders, the literature points to the existence of a high heterogeneity of the clinical presentation, which might indicate that we are also dealing with a spectrum of disorders. In the latest literature, it is indicated that there are as many as eight types of BD, which, although having some common elements—primarily the dominance of symptoms of mood disorders—can differ significantly in their picture and, above all, probably have a diverse etiopathological background. The understanding of their basis has evolved along with diagnostic concepts, especially in the field of the genetics of psychiatric disorders. The majority of mental disorders are linked to a wide variety of genes that show multiple mutations and/or polymorphisms that show significant associations with the risk of disease—or worsening symptoms. For this reason, the authors indicate that the risk of bipolar affective disorder, as well as its clinical picture, may be related to, firstly, the mass effect in the number of pro-disease alleles (a quantitative factor indicating the risk of obtaining a diagnosis) in the genome and, secondly, to the genes in which these polymorphisms occur in a given patient (a qualitative factor determining the clinical presentation). Thus, research on the biochemistry and genetics of psychiatric disorders must consider that the analyzed parameters may be responsible for only some of the symptoms of the disease—selected elements of the clinical picture—as well as that they may occur only in selected patients. For this reason, analyses detailing different groups of symptoms and exploring them in the context of the examined parameters are interesting, as they allow them to be linked and, for example, potential phenotypes of bipolar affective disorder to be selected depending on the patient’s genetic profile.

In our study, the occurrence of types I and II BD was associated with lower concentrations of nesfatin-1 (compared to the concentrations of neuropeptides in the group of undiagnosed people). In addition, in the logistic regression analysis, a decrease in nesfatin-1 was a strong risk factor for BD diagnosis. In the currently available literature, there are isolated reports on the relationship between nesfatin-1 and mood disorders. For example, in a study by Emula et al. among males with mania, significantly lower levels of nesfatin-1 were revealed than in a healthy control group, which is consistent with the results of our study. It has also been observed that electroconvulsive therapy with antipsychotic treatment, as well as an antipsychotic treatment alone, does not have a significant effect on nesfatin-1 levels after treatment of a manic episode [34]. In addition, there are two more studies on affective disorders available in the literature, but they focus only on unipolar depression. Xia et al. reported higher plasma concentrations of nesfatin-1 in patients with major depressive disorder, which they believe may be related to corticosterone, IL-6 and CRP concentrations [35]. In contrast, a 2023 study of Chinese adolescents noted that plasma concentrations of nesfatin-1 increased with the severity of depression in adolescents. It even considered the concentration of this neuropeptide as a biomarker for depression severity, and the need for further studies in this area was recognized [23]. Therefore, the results of our study among people with BD are not similar to those recorded in people with depression, quite the opposite—lower concentrations were observed in people with BD than in the control group. Perhaps the concentration of nesfatin-1 could differentiate unipolar depression from BD in the future. However, this requires further research and a larger study population.

In the literature, nesfatin-1 is primarily described as an anorexigenic neuropeptide, which may potentiate the effects of PNX-14 on the release of reproductive hormones, such as LH, follicle-stimulating hormone and testosterone, in male rats [12,19]. Interestingly, among the studied young people with BD, there were patients not only with decreased and increased appetites but also without changes in appetite, and no correlation was found between the symptoms of appetite disorders and the concentration of nesfatin-1 in the patients’ serum. Due to the lack of analysis of the concentration of nesfatin-1 in 12 patients who had their blood drawn twice, it was unfortunately not possible to assess changes in the serum concentrations of this neuropeptide as a result of the use of pharmacotherapy, which has a significant effect on both the appetite and weight gain of patients. Therefore, it is difficult to ascertain whether the concentration of nesfatin-1 in patients with BD can affect their appetite.

The ”nalyzed patients in our study had an average of 2.09 (95% CI: 1.68–2.5) assessed productive symptoms. In the Sahpolat and Ari study, patients had lower mean nesfatin-1 levels during the first episode of psychosis (0.60 ± 1.00 ng/mL) than controls (0.75 ± 1.07 ng/mL), but this was not a statistically significant difference. There was also no statistically significant correlation between the concentration of nesfatin-1 in plasma and the total PANSS results in the group of patients (r = −0.262, *p* = 0.148) [36]. Thus, these authors observed a certain trend between the groups that did not significantly translate into the severity of the clinical picture. Therefore, it would be interesting to look at the timeline to determine whether all the subjects developed schizophrenia after the first psychotic episode, or whether there were people with BD in this group whose first episode of the disease manifested with severe psychotic symptoms, and then compare the concentrations of nesfatin-1 in these groups with each other. Subsequently, the analysis of nesfatin-1 concentrations could be performed again, and it could be determined whether the results obtained were due to the presence of patients with BD in the study group. An argument for this type of hypothesis is the results of subsequent studies evaluating the concentrations of nesfatin-1 and ghrelin in patients with schizophrenia, who had significantly higher serum levels of nesfatin-1 than a healthy control group. The serum concentration of nesfatin-1 was also significantly higher in schizophrenic patients with multiple sclerosis (10.51–350.8 pg/mL) than in the healthy control group (4.86–68.91 pg/mL) [37]. According to the researchers, the presence of multiple sclerosis contributed to the significantly higher levels of nesfatin-1 among people with schizophrenia. The authors of the publication postulated that nesfatin-1 may play a role in new research on the treatment of schizophrenia and its metabolic effects [37]. These results are different from those observed in our study in children and adolescents with BD, who often have psychotic symptoms during exacerbations of the disease, as the concentrations of nesfatin-1 were lower than in the control group.

In addition, a study in children and adolescents with BD types I and II reported higher SPX concentrations, as well as an increase in SPX after 6 weeks of treatment. According to the available literature, SPX in animal models reduces insulin secretion and has, inter alia, antidepressant effects [38]. The statistically significant negative correlation between the number of manic symptoms and this neuropeptide in the current study, in the context of the available literature, points to the necessity of a profound analysis of its effects in people with BD. A study on rats conducted by Pałasz et al. in 2016 showed that a prolonged intraperitoneal administration of escitalopram (an antidepressant) resulted in an increased expression of the SPX gene in the hippocampus and striatum, while expression in the hypothalamus was decreased [39]. In another study, a prolonged intraperitoneal administration of haloperidol and chlorpromazine increased the expression of SPX mRNA and proopiomelanocortin (POMC) in the amygdala of rats [40]. Previous animal studies on the effect of SPX on anxiety and depression indicate that it is related to the CRH system, while the CRH system itself is connected to the serotonergic system [41,42,43]. It is thought that SPX-producing neurons are often in close proximity to serotonergic (5-HT) neuron fibers, which also adds to theories about the SPX-CRH-5-HT interaction and, thus, the possible effects of SPX on mood and behavior. In the context of previous reports on the antidepressant and anxiolytic effects of SPX, the results obtained in our study require further analysis, as they do not confirm that this neuropeptide plays such a role in BD.

It should be noted that the statistical significance of the differences In the level of SPX between the study and control groups diminished after the Bonferroni correction. However, in this case, applying this correction is very likely to have erroneously dismissed this result as non-statistically significant. In the analysis of this type of statistical procedure, one should always take into account the danger and imperfection of the Bonferroni correction suggested in the literature, which significantly increases the risk of erroneously rejecting hypotheses about the existence of relationships between variables. When performing multiple comparisons, in addition to the significance level determined using the Bonferroni correction, it is necessary to consider the obtained significance level values, the size of the difference between the means and the ranges of confidence intervals, and the number of statistically significant results obtained. In the case of SPX, we observed very low *p*-values, combined with a large difference between the mean, non-overlapping confidence intervals and a result that appears reliable based on the available literature. These factors significantly reduce the risk of randomness of the observed relationship and, consequently, indicate that the application of the Bonferroni amendment was unnecessary.

The results of the obtained SPX concentrations after 6 weeks of treatment are also puzzling, where an increase in the concentration of the neuropeptide was noted among 12 patients with a simultaneous improvement in mental state and a decrease in the symptoms of BD. At the same time, it should be considered to what extent this is a natural variability of this neuropeptide (as well as PNX) over time, the effect of an improving mental state, the disappearance of BD symptoms or the effect of pharmacological treatment. Treatment with quetiapine (a mood stabilizer) in a study by Nikisch et al. affected the level of NPY-LI (like immunoreactivity) in cerebrospinal fluid [44]. However, in another study conducted on rats, olanzapine decreased the expression of NPQ/SPX mRNA in the brainstem and increased the level of SMIM20/PNX mRNA [45]. However, at this point, it should be emphasized once again that the analyzed group was very small, and after the Bonferroni correction, the results lost their statistical significance.

Interestingly, in the case of PNX, there were no statistically significant differences between the study group and the control group, but an increase in its level was observed after 6 weeks of treatment. PNX is a neuropeptide detected in central and peripheral human tissues that has been linked to anxiety levels. A study conducted among men with obesity showed that PNX levels were negatively associated with anxiety [17]. However, it should be noted that, in the case of BD in the pediatric population, anxiety symptoms are frequent and are also considered a predictor of BD development in this age group [46]. In addition, anxiety disorders (i.a., panic disorder, agoraphobia, generalized anxiety disorder, and post-traumatic stress disorder) are comorbid in 54% of children and adolescents with BD, and their comorbidity with BD seems to be associated with poorer functioning of patients [47]. Therefore, it should be considered that anxiety symptoms may be frequent in a group of subjects hospitalized due to BD. Further studies (due to the relationship between PNX and anxiety described in the publications) should therefore aim to measure serum neuropeptide concentrations with a simultaneous assessment of the severity of anxiety symptoms in the examined subjects.

The literature also draws attention to the potential effect of PNX on food Intake [48], which requires further research, with particular emphasis on the relationship between the PNX concentrations and body weight/BMI of the examined subjects. It has been described to have anti-inflammatory and cell-protecting effects; affect behavior; and participate in sensory perception, memory processes and energy metabolism. In addition to the central nervous system, PNX is also expected to affect the heart, ovaries, adipose tissue and pancreatic islets. In addition, PNX is believed to play an important role in the female reproductive system, where it increases luteinizing hormone secretion, stimulates oocyte maturation and increases the number of ovulated oocytes [49]. Thus, it may be necessary to examine whether gender can have an impact on its concentration in serum and take this into account when drawing conclusions from the conducted study, where girls dominate in the study group and boys dominate in the control group.

In our study, kisspeptin only showed statistically significant differences in concentrations depending on the episode of BD. In animal models and humans, kisspeptin modulates glucose-stimulated insulin secretion, as well as food intake and energy expenditure. In animals, it is involved in the control and behavior of reproduction, and in humans, it is involved in the modulation and sexual and emotional processing of the brain [32]. Kisspeptin is also considered a key regulator of puberty and fertility [50]. In addition, it is believed to have antidepressant and fear-suppressing effects [32], which seems interesting in the context of the obtained elevated concentrations of kisspeptin in the study group during depressive episodes, while there was no statistically significant difference after 6 weeks of BD treatment. Hofmann et al. 2017 observed that kisspeptin positively correlates with BMI and tissue and fat mass in patients with anorexia nervosa, but they did not note any association with depression or anxiety [51]. In other studies, kisspeptin-13 (one of the endogenous isoforms, consisting of 13 amino acids) stimulated the hypothalamic–pituitary–adrenal axis (HPA) after its administration into the ventricles and therefore induced hyperthermia and motor behavior in rats. In mice, interaction with 5-HT2 receptors resulted in antidepressant-like effects. Interestingly, selective antagonists of serotonin α2-adrenergic and 5-HT2 receptors (phenoxybenzamine, yohimbine and cyproheptadine) abolished the behavioral effects of kisspeptin-13—according to the researchers, this may suggest that the antidepressant properties of kisspeptin-13 are due to interactions with the adrenergic system and serotonergic signaling [52,53]. At this point, it should also be noted that, after the Bonferroni correction, the *p*-value for the difference in kisspeptin concentrations between episodes lost its statistical significance. However, taking into account the large difference in the absolute values in the mean values and the size of the study population, this is a result that, although subject to the risk of randomness due to multiple comparisons, clearly indicates the existence of this type of tendency in this population.

It should be noted that, in the study conducted at DPPDA, none of the analyzed neuropeptides was significantly correlated with the number of BD symptoms. Thus, it does not appear that the concentrations of SPX, PNX, nesfatin-1 or kisspeptin can be used as biomarkers for the severity of BD in the way that Sun et al. considered nesfatin-1 in depression in their 2023 study [23].

In the analysis of the results obtained, the limitations of the presented study should also be considered. First of all, attention should be paid to the disproportion in the sex distribution between the study and control groups, which, for example, in the case of nesfatin-1, may significantly affect the interpretation of the results due to the gender-dependent nature of the regulatory activity of this neuropeptide in the brain. This study also did not consider the effects of the following on the concentrations of the neuropeptides: comorbidities, pharmacotherapy used in the study participants before enrolment in the study group and additional drugs (except for mood stabilizers) taken during hospitalization. In the context of measuring concentrations at baseline and after 6 weeks of treatment, it should also be noted that measurements were only taken in the treatment group, and there was no reference to the untreated/control group. Furthermore, the number of patients taken into account was limited due to logistical possibilities and great difficulties in obtaining blood samples 6 weeks after recruitment. The average duration of hospitalization in our department is less than 6 weeks, and most parents refuse a follow-up visit. Therefore, the observed changes in neuropeptide concentrations may be due to their natural variability or other factors not considered in the analysis, the influence of which cannot be excluded at this time. Another issue is the fact that the presented analysis concerns the concentrations of neuropeptides in peripheral blood. This is a limitation that divides most of the papers in this field, and it results from the practical impossibility of studying cerebrospinal fluid, let alone brain tissues in the target group of patients, with the simultaneous undetermined relationships between the concentrations of the studied neuropeptides within the brain and in the peripheral blood.

Nevertheless, increased knowledge of the potential involvement of SPX, PNX, kisspeptin and nesfatin-1 in psychiatric disorders such as BD may help in the future development of therapeutic interventions targeting the signaling pathways of specific neuropeptides. While the results of some studies seem encouraging, there are still many unknowns. All possible applications of the neuropeptides studied, as well as their agonists and antagonists, are still in the realm of speculation. Therefore, further research is needed to determine their possible therapeutic potential in mental disorders [11,32].

## 5. Conclusions

Significantly higher concentrations of SPX and significantly lower concentrations of nesfatin-1 were observed in the study group. In the logistic regression analysis, a decrease in nesfatin-1 was found to be a strong risk factor for the diagnosis of BD. The concentration of kisspeptin was significantly higher in the group of patients with a depressive episode; however, none of the analyzed neuropeptides was significantly correlated with the number of BD symptoms. There was a statistically insignificant difference in the concentration of SPX in the group of patients with mania (lower concentration) and a statistically significant correlation with the number of manic symptoms of this neuropeptide (the more the symptoms, the lower the concentration).

## Figures and Tables

**Table 1:** Breakdown of assessed symptoms for statistical analysis.

Manic Symptoms	Depressive Symptoms	Productive Symptoms
Mood enhancement Agitation Shortening of nighttime sleep Appetite change Attention deficits Undertaking a wide variety of activities Taking risky behaviors Acceleration of the train of thoughts Increased sexual energy	Depressed mood Decreased energy/fatigue Decline in activity Loss of interest Prolongation of nighttime sleep Appetite change Anhedonia Suicidal thoughts and attempts Self-harm Hygiene negligence Withdrawal from peer relationships	Delusions Hallucinations of any modality Pseudohallucinations of any modality

**Table 2:** Concentrations of analyzed neuropeptides in the study and control groups; *p*-value of the Mann–Whitney U test. Bonferroni correction for multiple comparisons α = 0.006; **—indicates statistically significant result with Bonferroni correction; *—indicates statistically significant result without Bonferroni correction.

	Study Group (Mean; 95% CI)	Control Group (Mean; 95% CI)	*p*-Value
PNX	1.59; (1.38–1.79)	1.15; (1.1–1.2)	*p* = 0.21
Nesfatin-1	1.42; (1.38–1.46)	1.81; (1.5–2.11)	*p* < 0.00000001 **
SPX	365.1; (271.09–458.92)	116.7; (69.6–163.9)	*p* = 0.007 *
Kisspeptin	67.7; (18.6–116.75)	54.8; (39.2–70.4)	*p* = 0.31

**Table 3:** Concentrations of analyzed neuropeptides depending on the type of episode at the time of hospitalization; *p*-value of the Mann–Whitney U test. Bonferroni correction for multiple comparisons α = 0.006; *—indicates statistically significant result without Bonferroni correction.

	Manic Episode (Mean; 95% CI)	Depressive Episode (Mean; 95% CI)	Control Group (Mean; 95% CI)	*p*-Value DEP vs. Mania
PNX	1.69 (1.43–1.94)	1.47 (1.12–1.82)	1.15; (1.1–1.2)	*p* > 0.05
Nesfatin-1	1.42 (1.36–1.48)	1.42 (1.36–1.48)	1.81; (1.5–2.11)	*p* > 0.05
SPX	348.48 (238.32–458.64)	365.01 (188–541.9)	116.7; (69.6–163.9)	*p* > 0.05
Kisspeptin	38.9 (25.4–52.4)	130.77 (0–294.4)	54.8; (39.2–70.4)	*p* = 0.04 *

**Table 4:** Concentrations of analyzed neuropeptides before and after the 6-week pharmacotherapy period; Wilcoxon’s *p*-value pair test. Bonferroni correction for multiple comparisons α = 0.016.

	Beginning of Treatment (Mean; 95% CI)	After 6 Weeks (Mean; 95% CI)	*p*-Value
PNX	1.13 (0.86–1.39)	1.58 (1.21–1.94)	*p* = 0.012
SPX	660.14 (477.6–842.6)	814.32 (618.12–1010.44)	*p* = 0.03
Kisspeptin	217.7 (−97.2–532.6)	166.48 (−35.4–368.4)	*p* = 0.81

**Table 5:** Correlation matrix between the concentrations of the analyzed neuropeptides in the whole population; *p*-value for Spearman’s correlations with Bonferroni correction for multiple comparisons = 0.0066; n/s stands for “not significant”.

	Nesfatine-1	PNX	SPX	Kisspeptin
Nesfatine-1		n/s	n/s	n/s
PNX	r = −0.044409		n/s	n/s
SPX	r = 0.536088	r = −0.001385		n/s
Kisspeptin	r = 0.260870	r = −0.242148	r = 0.426915	

## Data Availability

The datasets used and/or analyzed during the current study are available from the corresponding author upon reasonable request.
